# Supplementary material for: Trehalose increases tomato drought tolerance, induces defenses, and increases resistance to bacterial wilt disease
Source: PLoS One. 2022 Apr 27;17(4):e0266254. doi: 10.1371/journal.pone.0266254 (PMC9045674; doi:10.1371/journal.pone.0266254)
Supplement: S1 Table — (PDF) [file pone.0266254.s001.pdf]

Supplemental Table 1A. Strains and primers used in this study

Strain	***Notes***	***Reference***
Wild type GMI1000	*Ralstonia solanacearum* phylotype one sequevar 18, isolated from tomato in French Guyana	(Boucher, Barberis et al. 1985)
GMI1000 Δ*treA*	GMI1000 trehalase mutant cannot degrade trehalose; Spectinomycin/streptomycin-resistant	(MacIntyre, Barth et al. 2019)
Primer Name	***Sequence***	***Reference***
loxA F	TGGTAGACCACCAACACGAA	(Milling, Babujee et al. 2011)
loxA R	GACCAAAACGCTCGTCTCTC	"
osmF	TGTACCACGTTTGGAGGACA	"
osmR	ACCAGGGCAAGTAAATGTGC	"
PR1b B F	TTGGTGACTGCGGGATGA	"
PR1b B R	GGCGGCGGCTAGGTT T	"
PR1a B F	GAGGGCAGCCGTGCAA	"
PR1a B R	CACATTTTTCCACCAACACATTG	"
GluA F	TCA GCA GGG TTG CAA AAT CA	"
GluA R	CTCTAGGTGGGTAGGTGTTGGTTAA	"
Pin2 F	TGATGCCAAGGCTTGTACTAGAGA	"
Pin2 R	AGCGGACTTCCTTCTGAACGT	"
ACO5 F	AGATGGGCATTGGGTGAACA	"
ACO5 R	TTCAGCCATCACTCGGTGTC	"
DnaJ F	ATGAAGCGCCAGATACCATC	"
DnaJ R	TCAAGGCTCAATGTGTGCTC	"
Actin F	TCAGCAACTGGGATGATATG	"
Actin R	TTAGGGTTGAGAGGTGCTTC	"
rd22_F	ACGTGGCGTTATTTTTCCTG	(Yamaguchi-Shinozaki and Shinozaki 1993)
rd22_R	ATCTCCGGCATCTTCTCTGA	"
dhn_tas_F	CACCATGAGGGGCAACAGCA	(Kissoudis, Seifi et al. 2016)
dhn_tas_R	TCACCTTCATGTTGTCCAGGCATC	"
