# Supplementary material for: Trehalose increases tomato drought tolerance, induces defenses, and increases resistance to bacterial wilt disease
Source: PLoS One. 2022 Apr 27;17(4):e0266254. doi: 10.1371/journal.pone.0266254 (PMC9045674; doi:10.1371/journal.pone.0266254)
Supplement: S2 Table — (PDF) [file pone.0266254.s002.pdf]

Supplemental Table 1B. Concentration of hormones, oxylipins, and phenylpropanoids in tomato xylem sap following various treatments

Compound (nM)[a]	Healthy Avg[b]	WT avg[c]	WT FC[d]	WT p-val[e]	6 hr tre avg[f]	6 hr tre FC	6 hr tre p-val	24 hr tre avg[g]	24 hr tre FC	24 hr tre p-val	48 hr tre avg[h]	48 hr tre FC	48 hr tre p-val
12,13-diHOM	0.83	1.30	2	0.04	1.26	2	0.27	2.69	3	0.00	1.50	2	0.02
12,13-Ep-9KOM	5.38	6.86	1	0.24	7.93	1	0.02	4.82	1	0.64	7.62	1	0.04
12OH-JA	1.43	1.70	1	0.63	5.21	4	0.30	1.38	1	0.84	1.17	1	0.06
12OH-JA-Ile	12.37	15.23	1	0.81	20.67	2	0.29	22.97	2	0.14	57.67	5	0.00
12-OPDA	35.86	47.89	1	0.44	50.75	1	0.43	138.72	4	0.00	91.84	3	0.01
13-HOD	4.66	8.52	2	0.00	5.09	1	0.66	9.02	2	0.01	5.49	1	0.14
13-KOD	4.50	10.78	2	0.01	6.27	1	0.31	9.72	2	0.00	5.81	1	0.12
13,12-KODA	0.27	0.75	3	0.34	0.11	0	0.37	4.89	18	0.00	0.22	1	0.80
13,12-KOMA	1.78	3.18	2	0.23	1.23	1	0.47	9.67	5	0.00	2.23	1	0.58
2-HOD	0.24	0.35	1	0.44	0.36	1	0.18	0.30	1	0.55	0.29	1	0.66
9,10,11-THOD	88.94	191.22	2	0.02	134.71	2	0.09	214.49	2	0.00	153.30	2	0.02
9,10,11-THOM	237.57	811.16	3	0.00	430.01	2	0.02	572.57	2	0.00	379.05	2	0.00
9,10,13-THOD	3.47	4.47	1	0.28	9.91	3	0.00	5.78	2	0.00	6.06	2	0.02
9,10,13-THOM	62.08	100.56	2	0.03	147.39	2	0.00	128.47	2	0.00	96.87	2	0.01
9,10-diHOM	0.95	1.25	1	0.43	1.48	2	0.52	3.64	4	0.00	1.02	1	0.86
9,12,13-THOD	39.77	50.62	1	0.18	43.68	1	0.56	84.76	2	0.00	60.09	2	0.02
9,12,13-THOM	127.69	231.37	2	0.01	153.78	1	0.36	305.89	2	0.00	179.49	1	0.04
9-HOD	6.15	21.29	3	0.01	10.27	2	0.02	14.60	2	0.00	8.64	1	0.17
9-HOT	3.04	9.78	3	0.03	5.02	2	0.16	9.75	3	0.00	4.87	2	0.12
9-KOD	4.35	8.90	2	0.15	6.60	2	0.34	19.32	4	0.00	12.16	3	0.00
9-KOT	1.06	3.68	3	0.17	5.51	5	0.05	7.61	7	0.00	3.90	4	0.05
9OH-10KOM	8.81	5.36	1	0.07	10.81	1	0.15	9.05	1	0.88	8.97	1	0.92
9OH-12KOD	0.20	0.13	1	0.51	0.27	1	0.53	0.69	4	0.00	0.34	2	0.02
9OH-12KOM	1.88	2.48	1	0.03	2.96	2	0.24	3.97	2	0.00	2.11	1	0.36
9-oxo-NA	13.94	17.47	1	0.37	16.53	1	0.56	16.06	1	0.51	17.25	1	0.32
ABA	8.20	49.59	6	0.03	5.71	1	0.60	40.37	5	0.00	9.72	1	0.73

Compound (nM)[a]	Healthy Avg[b]	WT avg[c]	WT FC[d]	WT p-val[e]	6 hr tre avg[f]	6 hr tre FC	6 hr tre p-val	24 hr tre avg[g]	24 hr tre FC	24 hr tre p-val	48 hr tre avg[h]	48 hr tre FC	48 hr tre p-val
AZA	14.08	19.39	1	0.11	19.21	1	0.00	20.44	1	0.00	18.03	1	0.01
BA	7.18	12.37	2	0.13	11.34	2	0.19	13.29	2	0.00	9.74	1	0.02
CA	7.49	5.19	1	0.52	5.30	1	0.56	22.69	3	0.00	14.19	2	0.16
COUMA	2.04	149.10	73	0.05	2.42	1	0.83	5.22	3	0.11	0.00	0	0.12
dnOPDA	0.12	0.21	2	0.25	0.26	2	0.02	0.53	4	0.00	0.44	4	0.00
IAA	3.26	36.76	11	0.03	2.84	1	0.59	4.01	1	0.29	2.20	1	0.09
JA	16.71	9.42	1	0.22	26.64	2	0.33	49.41	3	0.01	71.36	4	0.00
JA-Ile	26.59	3.67	0	0.09	35.48	1	0.62	60.08	2	0.04	103.68	4	0.00
OPC-4	0.31	0.53	2	0.09	0.52	2	0.13	1.09	4	0.00	1.05	3	0.00
p12,13-DiHOD*	0.16	0.91	6	0.00	0.85	5	0.05	0.82	5	0.00	0.18	1	0.69
p9,10-DiHOD*	0.63	1.75	3	0.02	1.48	2	0.04	1.73	3	0.01	0.91	1	0.42
p9OH-TAN*	3.62	3.35	1	0.62	3.36	1	0.71	8.86	2	0.00	4.07	1	0.46
pOTD*	0.47	0.42	1	0.72	0.52	1	0.71	1.19	3	0.00	0.36	1	0.33
SA	67.53	31.73	0	0.21	55.50	1	0.69	173.44	3	0.01	55.09	1	0.65
TA	6.03	10.56	2	0.04	7.94	1	0.19	17.41	3	0.00	7.35	1	0.23
TAN	0.54	0.77	1	0.32	0.53	1	0.91	0.74	1	0.01	0.60	1	0.51

[a] Compound color key: Blue fill= 13-LOX pathway derived compound; Red fill= 9-LOX pathway derived compound; Purple fill= phenylpropanoid pathway derived compound; Gray fill= other; metabolites were measured with LC/MS/MS

[b] Xylem sap harvested from mock-inoculated/treated healthy BB plants

[c] Sap harvested from DI=1 *Rs* infected plants

[d] Fold-change values ≥ 2 are shaded in light red with dark red text.

[e] P-value derived from t-test of treatment compared to healthy; *P*<0.5 was shaded yellow with darker yellow text.

[f-g] Xylem sap harvested from uninoculated, trehalose treated plants 6, 24, and 48 h post-treatment
