# Supplementary material for: Trehalose increases tomato drought tolerance, induces defenses, and increases resistance to bacterial wilt disease
Source: PLoS One. 2022 Apr 27;17(4):e0266254. doi: 10.1371/journal.pone.0266254 (PMC9045674; doi:10.1371/journal.pone.0266254)
Supplement: S3 Table — (PDF) [file pone.0266254.s003.pdf]

Supplemental Table 1C. Metabolite key for hormone analysis

Q1[a]	Q3[b]	RT[c]	Metabolite	Formal Name
313.3	183.3	10.16	12,13-DiHOM	threo-12,13-dihydroxy-9(*Z*)-octadecenoic acid
309.4	208.9	12.46	(*E*)-KODA	12,13-epoxy-9-oxo-10(*E*)-octadecenoic acid
225	59	2.44	12OH-JA	12-hydroxy-jasmonic acid
338.3	130.1	3.19	12OH-JA-Ile	12-hydroxy-jasmonic acid isoluecine
291.3	165	11.13	12-OPDA	12-oxo-10(*Z*),15(*Z*)-phytodienoic acid
295.3	195.2	13.46	13-HOD	13(*S*)-hydroxy-9(*Z*),11(*E*)-octadecatrienoic acid
293.2	113.2	14.16	13-KOD	13-oxo-9(*Z*),11(*E*)-octadecadienoic acid
309.4	180.8	10.32	13,12-KODA	13-hydroxy-12-oxo-9(*Z*),15(*Z*)-octadecadienoic acid
311.3	194.9	11.42	13,12-KOMA	13-hydroxy-12-oxo-9(*Z*)-octadecenoic acid
293.2	191.1	15.15	2-HOD	2-hydroxy-9(*Z*),15(*Z*)-octadecadienoic acid
327.1	137	5.57	9,10,11-THOD	9(*S*),10(*S*),11(*R*)-trihydroxy-12(*Z*),15(*Z*)-octadecadienoic acid
329.5	201	6.69	9,10,11-THOM	9(*S*),10(*S*),11(*R*)-trihydroxy-12(*Z*)-octadecenoic acid
327.1	137	4.94	9,10,13-THOD	9(*S*),10(*S*),13(*S*)-trihydroxy-11(*E*),15(*Z*)-octadecadienoic acid
329.5	139	5.8	9,10,13-THOM	9(*S*),10(*S*),13(*S*)-trihydroxy-11(*E*)-octadecenoic acid
313.2	201.2	10.46	9,10-DiHOM	threo-9,10-dihydroxy-12(*Z*)-octadecenoic acid
326.9	229.1	5	9,12,13-THOD	9(*S*),12(*S*),13(*S*)-trihydroxy-10(*E*),15(*Z*)-octadecadienoic acid
329.5	210.9	5.74	9,12,13-THOM	9(*S*),12(*S*),13(*S*)-trihydroxy-10(*E*)-octadecenoic acid
295.3	171.1	13.53	9-HOD	9(*S*)-hydroxy-10(*E*),12(*Z*)-octadecadienoic acid
293.2	171.2	12.04	9-HOT	9(*S*)-hydroxy-10(*E*),12(*Z*),15(*Z*)-octadecatrienoic acid
293.2	195.1	12.25	9-KOD	9(*S*)-oxo-10(*E*),12(*Z*)-octadecatrienoic acid
291.3	184.7	13.04	9-KOT	9(*S*)-oxo-10(*E*),12(*Z*),15(*Z*)-octadecatrienoic acid
311.3	293.2	11.76	9,10-KOMA	9-hydroxy-10-oxo-12(*Z*)-octadecadienoic acid
309.1	291.3	9.02	9,12-KODA	9-hydroxy-12-oxo-10(*E*),15(*Z*)-octadecadienoic acid
311.3	293.1	10.29	9,12-KOMA	9-hydroxy-12-oxo-10(*E*)-octadecenoic acid
263.4	153.1	3.33	ABA	Abscisic acid
186.9	124.9	2.83	AZA	Azelaic Acid
121.1	76.9	2.54	BA	Benzoic acid
147	102.9	3.6	CA	Cinnamic acid
162.9	118.9	2.05	COUMA	Coumaric acid
263.1	165	8.29	dn-12-OPDA	dinor-12-oxo-8(*Z*),13(*Z*)-phytodienoic acid
174	129.9	2.77	IAA	Indole-3-acetic acid
209.1	59	4.24	JA	(+)-7-iso-jasmonic acid
322.2	130	6.82	JA-Ile	(+)-7-Jasmonoyl-L-isoleucine
237	165.1	6.38	OPC-4	8-[3-oxo-2-cis-[(*Z*)-2-pentenylcyclopentyl]butanoic acid
311.2	183.2	10.3	12,13-DiHOD*	threo-12,13-dihydroxy-9(*Z*),15(*Z*)-octadecenoic acid
311.2	201.2	10.35	9,10-DiHOD*	threo-9,10-dihydroxy-12(*Z*),15(*Z*)-octadecenoic acid
226.9	208.7	3.2	9OH-TAN*	9-hydroxy-12-oxo-10(*E*)-dodecenoic acid
223	195	7.3	OTD*	13-oxo-9(*Z*),11(*E*)-tridecadienoic acid
137	92.9	2.45	SA	Salicylic acid
226.9	182.7	5.36	TA	Traumatic acid
210.9	182.7	6.56	TAN	Traumatin

[A-B] Select m/z ions for compound identification (see Methods section)
[C] Compound retention time
