# Supplementary material for: Trehalose increases tomato drought tolerance, induces defenses, and increases resistance to bacterial wilt disease
Source: PLoS One. 2022 Apr 27;17(4):e0266254. doi: 10.1371/journal.pone.0266254 (PMC9045674; doi:10.1371/journal.pone.0266254)
Supplement: S1 Fig — Growth of Rs cultured in rich CPG broth supplemented with ABA in DMSO, or water with DMSO because ABA stock solutions were dissolved in DMSO (100 μM treatments contained 0.1 μL of either DMSO or ABA stock solution, and 50 μM treatments contained 0.05 μL DMSO or ABA stock solution) (ANOVA Area Under Curve/AUC, Fisher’s LSD multiple comparisons to CPG control, 100 μM DMSO, P = .55; 50 μM DMSO, P = .53; 100 μM ABA, P = .67; 50 μM ABA, P = .45). Growth was measured spectrophotometrically using a Bio-Tek plate reader; the data represent eight technical reps/treatment. The bars represent the standard error of the mean. (PDF) [file pone.0266254.s004.pdf]

MacIntyre et al **Trehalose increases tomato drought tolerance, induces defenses, and increases resistance to bacterial wilt disease**

Supplemental Figure 1

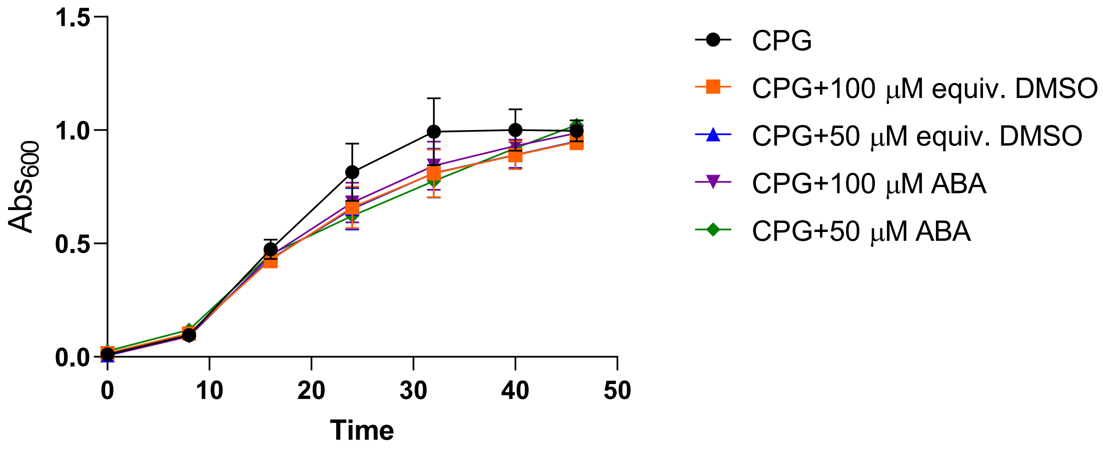

Supplemental Figure 1. ABA and DMSO are not toxic to *Rs*. Growth of *Rs* cultured in rich CPG broth supplemented with ABA in DMSO, or water with DMSO because ABA stock solutions were dissolved in DMSO (100 µM treatments contained 0.1 µL of either DMSO or ABA stock solution, and 50 µM treatments contained 0.05 µL DMSO or ABA stock solution) (ANOVA Area Under Curve/AUC, Fisher's LSD multiple comparisons to CPG control, 100 µM DMSO, *P*=.55; 50 µM DMSO, *P*=.53; 100 µM ABA, *P*=.67; 50 µM ABA, *P*=.45). Growth was measured spectrophotometrically using a Bio-Tek plate reader; the data represent eight technical reps/treatment. The bars represent the standard error of the mean.
