# Supplementary material for: Trehalose increases tomato drought tolerance, induces defenses, and increases resistance to bacterial wilt disease
Source: PLoS One. 2022 Apr 27;17(4):e0266254. doi: 10.1371/journal.pone.0266254 (PMC9045674; doi:10.1371/journal.pone.0266254)
Supplement: S2 Fig — A) Water efficiency, measured with a LI-COR as photosynthetic rate/transpiration, in trehalose treated plants compared to water only controls (t-test, P<0.5). B) Photosynthesis, measured as Anet, in trehalose treated plants (Mixed model ANOVA, Tukey’s HSD, P>.05). The water efficiency and photosynthetic data represent 20 plants/treatment. LI-COR measurements were taken ~10 min after treatment and then in the morning every time point after. Shaded bars on the graphs represent night periods. All data were calculated from the same LICOR time points. The bars on the data points represent the standard deviation. C) Relative water content of leaves was calculated from the fresh weight, hydrated weight, and dry weight of leaves from water control or trehalose treated BB tomatoes (t-test, P = .14). The data represent two biological replicates with five technical replicates (leaves)/treatment). The bars represent the standard deviation. D) ‘Bonny Best’ tomato seedlings were placed in root growth pouches, watered with 10mL of either water or trehalose for one day, then watered as needed for ten days. Root length was measured after ten days (t-test, P = .31). The data represent three biological replicates with ten roots/treatment. Percent of new root growth was calculated as %new root growth = (new growth/total root length)*100. The bars represent the standard deviation. E) SPAD meter measurements of chlorophyll content in leaves following trehalose treatment (2-way ANOVA Fishers LSD multiple comparison, * = P<0.5 or lower). The bars represent the standard deviation. (PDF) [file pone.0266254.s005.pdf]

MacIntyre et al **Trehalose increases tomato drought tolerance, induces defenses, and increases resistance to bacterial wilt disease**

Supplemental Figure 2

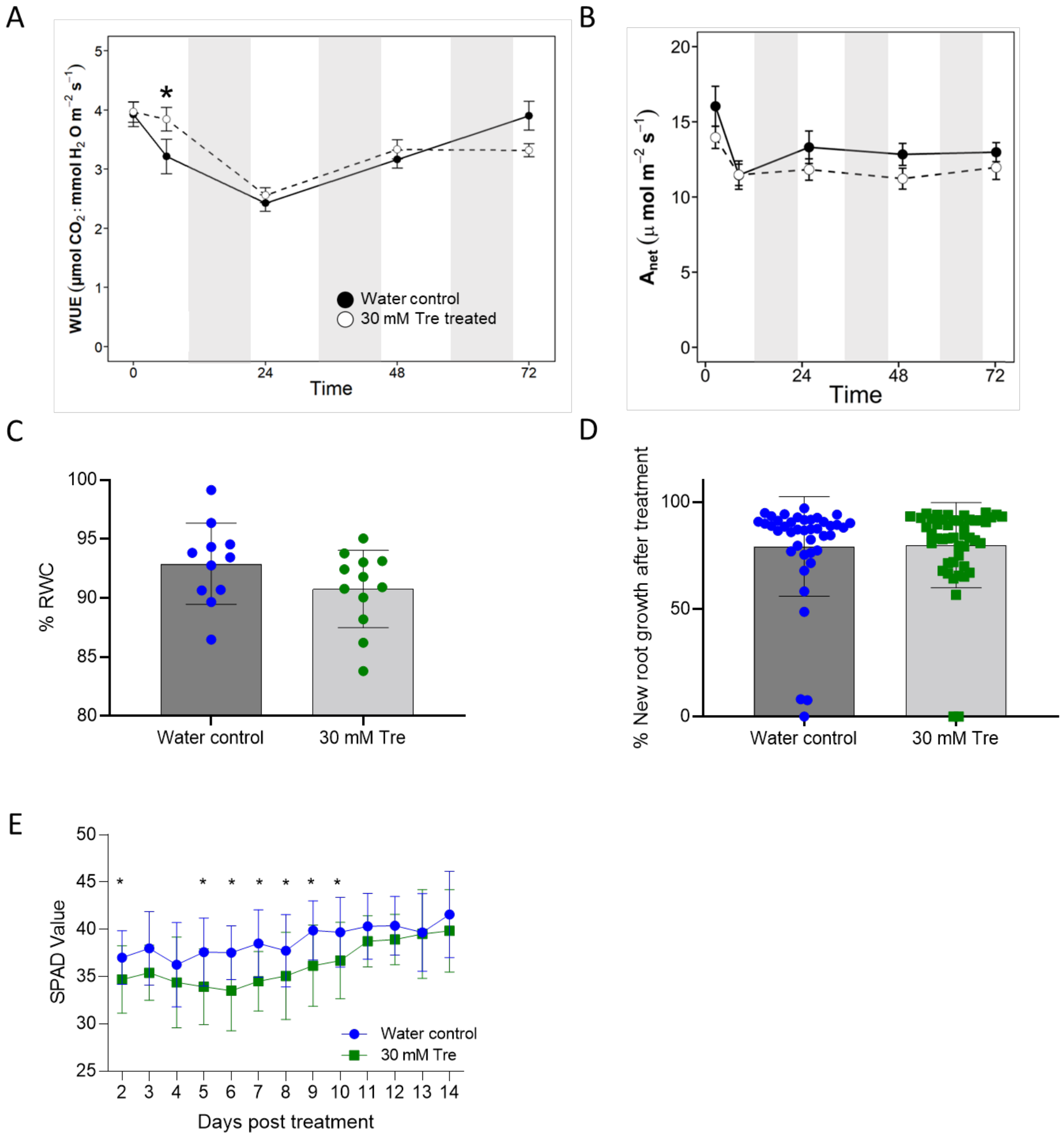

Supplemental Figure 2. Trehalose treatment temporarily improves water efficiency without affecting photosynthesis; trehalose treatment does not affect root growth and relative water content of leaves but lowers chlorophyll concentration. A) Water efficiency, measured with a LI-COR as photosynthetic rate/transpiration, in trehalose treated plants compared to water only controls (t-test, *P*<0.5). **B)** Photosynthesis, measured as A_{net}, in trehalose treated plants (Mixed model ANOVA, Tukey's HSD, *P*>.05). The water efficiency and photosynthetic data represent 20 plants/treatment. LI-COR

measurements were taken ~10 min after treatment and then in the morning every time point after. Shaded bars on the graphs represent night periods. All data were calculated from the same LICOR time points. The barson the data points represent the standard deviation. **C)** Relative water content of leaves was calculated from the fresh weight, hydrated weight, and dry weight of leaves from water control or trehalose treated BB tomatoes (t-test, P=.14). The data represent two biological replicates with five technical replicates (leaves)/treatment). The bars represent the standard deviation. **D)** 'Bonny Best' tomato seedlings were placed in root growth pouches, watered with 10mL of either water or trehalose for one day, then watered as needed for ten days. Root length was measured after ten days (t-test, P=.31). The data represent three biological replicates with ten roots/treatment. Percent of new root growth was calculated as %new root growth= (new growth/total root length)*100. The bars represent the standard deviation. **E)** SPAD meter measurements of chlorophyll content in leaves following trehalose treatment (2-way ANOVA Fishers LSD multiple comparison, * = P<0.5 or lower). The bars represent the standard deviation.
