# Supplementary material for: Trehalose increases tomato drought tolerance, induces defenses, and increases resistance to bacterial wilt disease
Source: PLoS One. 2022 Apr 27;17(4):e0266254. doi: 10.1371/journal.pone.0266254 (PMC9045674; doi:10.1371/journal.pone.0266254)
Supplement: S3 Fig — A) Disease progress curve of plants treated with trehalose or water, then infected with either wild-type Rs or a ΔtreA mutant unable to catabolize trehalose (one-way ANOVA of areas under the curve, WT H20 vs. WT tre, P = .0008, ΔtreA H20 vs. ΔtreA tre, P = .017). The data represent three bioreps each containing 13–15 plants per treatment. The bars represent the standard error. B) Disease development in plants sprayed once with 30 mM trehalose or water, and then soil-soak inoculated with Rs 48 h later (ANOVA of AUC, Fisher’s LSD multiple comparisons to H20 soil, H20 spray, P = .67; tre spray, P = .63; tre soil, P = .63). The data represent three biological replicates each containing fifteen plants per treatment. The bars represent the standard error. C) Colonization of tomato leaves by the bacterial leaf spot pathogen Xanthomonas gardneri; plants were dip-inoculated with X. gardneri 72 h after root treatment with water or 30 mM trehalose (Mann-Whitney, day 3, P = .06; day 6, P = .61; day 9, P = .08). The data represent three biological replicates with twelve plants/treatment/timepoint. The graph displays the geometric means; bars indicate the geometric standard deviation of the data. (PDF) [file pone.0266254.s006.pdf]

MacIntyre et al **Trehalose increases tomato drought tolerance, induces defenses, and increases resistance to bacterial wilt disease**

Supplemental Figure 3

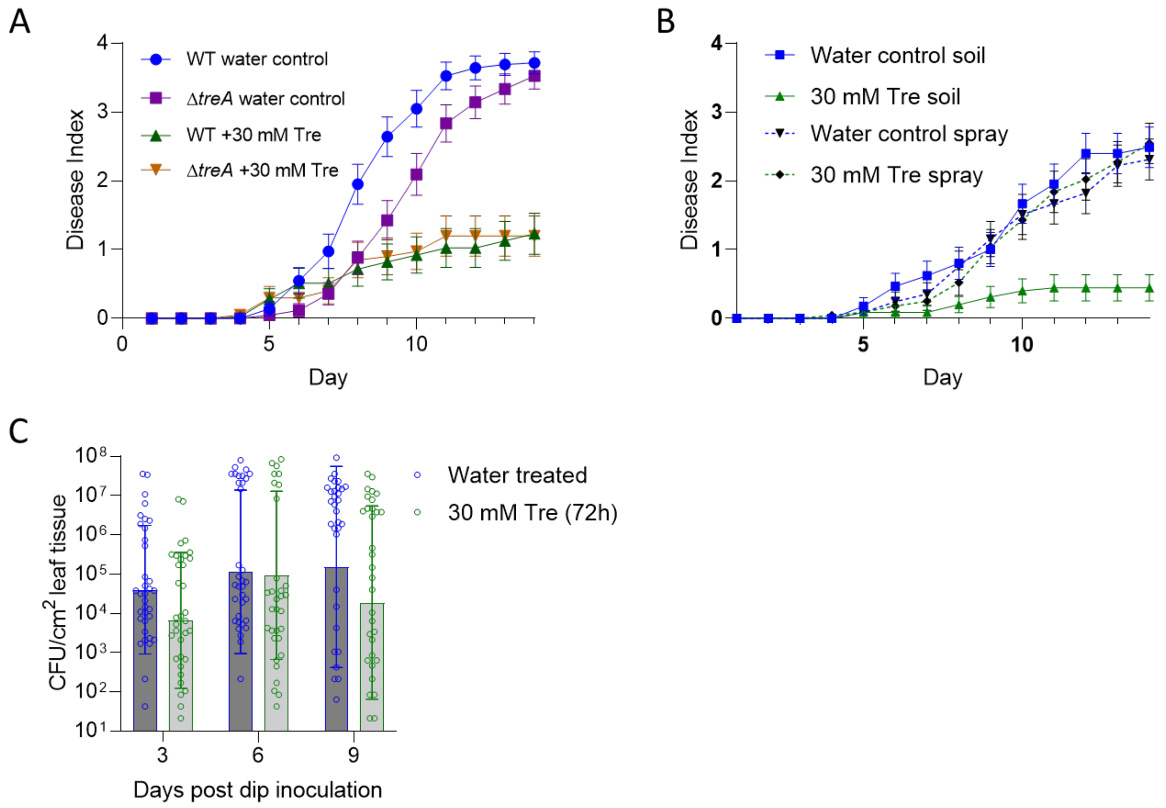

Supplemental Figure 3. Trehalose treatment does not nutritionally enrich the soil environment for *R. solanacearum*, and its protection is limited to root application. A) Disease progress curve of plants treated with trehalose or water, then infected with either wild-type *Rs* or a Δ*treA* mutant unable to catabolize trehalose (one-way ANOVA of areas under the curve, WT H_20 vs. WT tre, *P*=.0008, Δ*treA* H_20 vs. Δ*treA* tre, *P*=.017). The data represent three bioreps each containing 13-15 plants per treatment. The bars represent the standard error. **B)** Disease development in plants sprayed once with 30 mM trehalose or water, and then soil-soak inoculated with *Rs* 48 h later (ANOVA of AUC, Fisher's LSD multiple comparisons to H_20 soil, H_20 spray, *P*=.67; tre spray, *P*=.63; tre soil, *P*=.63). The data represent three biological replicates each containing fifteen plants per treatment. The bars represent the standard error. **C)** Colonization of tomato leaves by the bacterial leaf spot pathogen *Xanthomonas gardneri*; plants were dip-inoculated with *X. gardneri* 72 h after root treatment with water or 30 mM trehalose (Mann-Whitney, day 3, *P*=.06; day 6, *P*=.61; day 9, *P*=08). The data represent three biological replicates with

twelve plants/treatment/timepoint. The graph displays the geometric means; bars indicate the geometric standard deviation of the data.
