# Supplementary material for: Trehalose increases tomato drought tolerance, induces defenses, and increases resistance to bacterial wilt disease
Source: PLoS One. 2022 Apr 27;17(4):e0266254. doi: 10.1371/journal.pone.0266254 (PMC9045674; doi:10.1371/journal.pone.0266254)
Supplement: S4 Fig — DAB staining of ‘Bonny best’ tomato leaves infused with water, 109 CFU/mL Rs, or 30 mM trehalose solution to assess the effect of trehalose treatment on ROS production. The data represent three biological replicates, with four plants per biological replicate per treatment. Photos are representative samples and images were uniformly sharpened 25% to increase contrast. (PDF) [file pone.0266254.s007.pdf]

MacIntyre et al **Trehalose increases tomato drought tolerance, induces defenses, and increases resistance to bacterial wilt disease**

Supplemental Figure 4

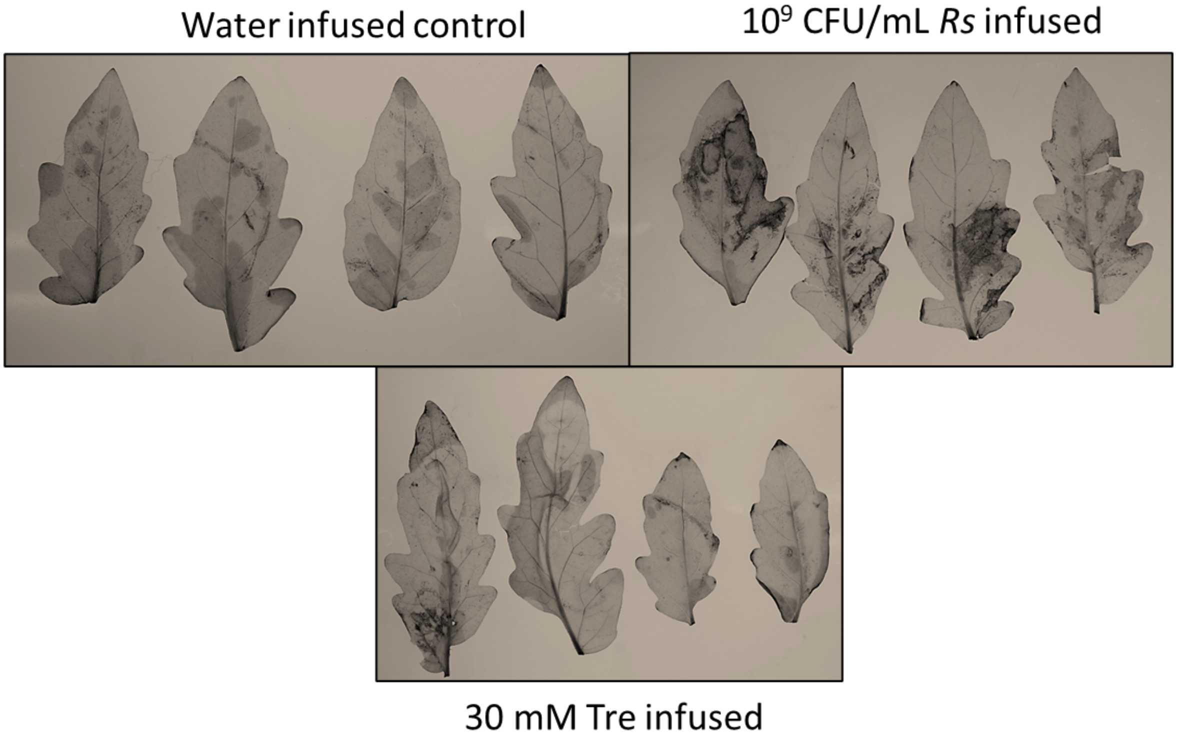

Supplemental Figure 4. Infusing leaves with trehalose did not trigger ROS production in 'Bonny Best' tomato leaves. DAB staining of 'Bonny best' tomato leaves infused with water, 10^9 CFU/mL *Rs*, or 30 mM trehalose solution to assess the effect of trehalose treatment on ROS production. The data represent three biological replicates, with four plants per biological replicate per treatment. Photos are representative samples and images were uniformly sharpened 25% to increase contrast.
