# Supplementary material for: Trehalose increases tomato drought tolerance, induces defenses, and increases resistance to bacterial wilt disease
Source: PLoS One. 2022 Apr 27;17(4):e0266254. doi: 10.1371/journal.pone.0266254 (PMC9045674; doi:10.1371/journal.pone.0266254)
Supplement: Attachment — Submitted filename: MacIntyre et al revised cover letter.docx [file pone.0266254.s008.docx]

March 10, 2022

College of Agricultural and Life Sciences	Russell Laboratories	University of Wisconsin-Madison

1630 Linden Drive	Madison, Wisconsin 53706	608/262-1410	Fax: 608/263-2626	www.plantpath.wisc.edu

Dr Ching-Hong Yang

Academic Editor, PLoS ONE

Dear Professor Yang,

Thank you and the reviewers for the rapid turnaround of our manuscript, PONE-D-22-04449 “Trehalose increases tomato drought tolerance, induces defenses, and increases resistance to bacterial wilt disease”, by MacIntyre et al. We understand that reviewing and editing are unpaid labor for the scientific community. It is much appreciated.

We have revised and resubmitted the manuscript in accordance with your feedback. Below please find our responses (in red text) to the journal requirements and the reviewers’ comments. We hope this work will now be acceptable for publication in PLoS ONE.

Sincerely,

Caitilyn Allen

Ethyl and O.N. Allen Professor

Department of Plant Pathology

[callen@wisc.edu](mailto:callen@wisc.edu)

608-556-3369 (cell)

PONE-D-22-04449 “Trehalose increases tomato drought tolerance, induces defenses, and increases resistance to bacterial wilt disease”, by MacIntyre et al.

**Responses to Reviews and Journal requirements**

1.Please consider the comments of the reviewers regarding the labelling of figures, carefully proofread the manuscript and check for correct labelling/copyediting.

-Thank you, copyediting has been completed and reviewer suggestions addressed.

2. Please review your reference list to ensure that it is complete and correct. If you have cited papers that have been retracted, please include the rationale for doing so in the manuscript text, or remove these references and replace them with relevant current references. Any changes to the reference list should be mentioned in the rebuttal letter that accompanies your revised manuscript. If you need to cite a retracted article, indicate the article’s retracted status in the References list and also include a citation and full reference for the retraction notice.

-References have been reformatted to the Vancouver style. Species names have been italicized where needed (ref. 84, 85, 86).

-Supplemental tables and figures now have their own reference list in the Vancouver style.

- The citation list has been run through the Zotero/Retraction Watch creen and, to the best of our knowledge, none of these papers have been retracted.

**Comments to the Author**

1. Is the manuscript technically sound, and do the data support the conclusions?

The manuscript must describe a technically sound piece of scientific research with data that supports the conclusions. Experiments must have been conducted rigorously, with appropriate controls, replication, and sample sizes. The conclusions must be drawn appropriately based on the data presented.

Reviewer #1: Yes

Reviewer #2: Yes

2. Has the statistical analysis been performed appropriately and rigorously?

Reviewer #1: Yes

Reviewer #2: Yes

3. Have the authors made all data underlying the findings in their manuscript fully available?

The [PLOS Data policy](http://www.plosone.org/static/policies.action#sharing) requires authors to make all data underlying the findings described in their manuscript fully available without restriction, with rare exception (please refer to the Data Availability Statement in the manuscript PDF file). The data should be provided as part of the manuscript or its supporting information, or deposited to a public repository. For example, in addition to summary statistics, the data points behind means, medians and variance measures should be available. If there are restrictions on publicly sharing data—e.g. participant privacy or use of data from a third party—those must be specified.

Reviewer #1: Yes

Reviewer #2: Yes

4. Is the manuscript presented in an intelligible fashion and written in standard English?

PLOS ONE does not copyedit accepted manuscripts, so the language in submitted articles must be clear, correct, and unambiguous. Any typographical or grammatical errors should be corrected at revision, so please note any specific errors here.

Reviewer #1: Yes

Reviewer #2: Yes

5. Review Comments to the Author

Please use the space provided to explain your answers to the questions above. You may also include additional comments for the author, including concerns about dual publication, research ethics, or publication ethics. (Please upload your review as an attachment if it exceeds 20,000 characters)

Reviewer #1: The manuscript titled “Trehalose increases tomato drought tolerance, induces defenses, and increases resistance to bacterial wilt disease” studies the influence of treating trehalose on tomato. The manuscript describes a comprehensive study on the relationship of trehalose, Ralstonia solanacearim, plant defenses and drought tolerance of tomato plants. The work is well structured and is presented clearly. Techniques and methodologies are suitable and well described. Authors present results of original research and the rest of the requirements of the journal are fulfilled. I have just a few minor comments and suggestions for corrections:

Results
Line 261: Fig 3B should be corrected to Fig 3C. -Thank you, this has been corrected.
Line 264: Fig 3C should be corrected to Fig 3D. -Thank you, this has been corrected.
Supplemental Figure 3C, colonization of tomato leaves by the bacterial leaf spot pathogen Xanthomonas gardneri, …., day 9, p=08), the decimal point was missing. --Thank you, this has been corrected.

Discussion
If possible, insert the corresponding figure numbers at the paragraph for easily reading. For example, page 16, line 464-465: In response to R. solanacearum infection, tomatoes downregulated multiple aquaporins, suggesting that plant may try to alter water flow during bacterial wilt (Fig 1).

-Thank you for the suggestion. Figures asre now called out in the Discussion (see lines 464, 473, 459, 457, 488, 489, 498, 499, 509, 510-513, 527, 536, 550, 558, 565, 568, 578, 584-585, 588, 596, 599, 611, 618, and 639-640).

Reviewer #2: The authors describe the role of trehalose presented in xylem sap of Ralstonia solanacearum infected-tomato and confirm the link between plant responses to biotic and abiotic stress. All of these are consistent with their previous trehalose work in R. solanacearum. It is clear from this and previous works that plants perceive trehalose as a DAMP and respond with systemic disease resistance. In turn, R. solanacearum degrades trehalose as a counter-defense.

6. PLOS authors have the option to publish the peer review history of their article ([what does this mean?](https://journals.plos.org/plosone/s/editorial-and-peer-review-process#loc-peer-review-history)). If published, this will include your full peer review and any attached files.


If you choose “no”, your identity will remain anonymous but your review may still be made public.


**Do you want your identity to be public for this peer review?** For information about this choice, including consent withdrawal, please see our [Privacy Policy](https://www.plos.org/privacy-policy).

Reviewer #1: No

Reviewer #2: No

[NOTE: If reviewer comments were submitted as an attachment file, they will be attached to this email and accessible via the submission site. Please log into your account, locate the manuscript record, and check for the action link "View Attachments". If this link does not appear, there are no attachment files.]

While revising your submission, please upload your figure files to the Preflight Analysis and Conversion Engine (PACE) digital diagnostic tool, <https://pacev2.apexcovantage.com/>. PACE helps ensure that figures meet PLOS requirements. To use PACE, you must first register as a user. Registration is free. Then, login and navigate to the UPLOAD tab, where you will find detailed instructions on how to use the tool. If you encounter any issues or have any questions when using PACE, please email PLOS at [figures@plos.org](mailto:figures@plos.org). Please note that Supporting Information files do not need this step.

- Done, thank you.
